# Gold Nanoparticles Disrupt the IGFBP2/mTOR/PTEN Axis to Inhibit Ovarian Cancer Growth

**DOI:** 10.1002/advs.202200491

**Published:** 2022-09-14

**Authors:** Md. Nazir Hossen, Lin Wang, Shailendra Kumar Dhar Dwivedi, Yushan Zhang, Geeta Rao, Chandra Kumar Elechalwar, Vinit Sheth, Anindya Dey, Sima Asfa, Suresh Kumar Gulla, Chao Xu, Kar‐Ming Fung, J. David Robertson, Magdalena Bieniasz, Stefan Wilhelm, Resham Bhattacharya, Priyabrata Mukherjee

**Affiliations:** ^1^ Peggy and Charles Stephenson Cancer Center University of Oklahoma Health Science Center Oklahoma City Oklahoma 73104 USA; ^2^ Department of Pathology University of Oklahoma Health Science Center Oklahoma City Oklahoma 73104 USA; ^3^ Department of Pharmaceutical and Biomedical Sciences California Northstate College of Pharmacy Elk Grove CA USA; ^4^ Aging and Metabolism Research Program Oklahoma Medical Research Foundation Oklahoma City OK 73104 USA; ^5^ Department of Obstetrics and Gynecology University of Oklahoma Health Science Center Oklahoma City Oklahoma 73104 USA; ^6^ Stephenson School of Biomedical Engineering University of Oklahoma Norman Oklahoma 73019 USA; ^7^ Department of Biostatistics and Epidemiology Hudson College of Public Health University of Oklahoma Health Sciences Center Oklahoma City Oklahoma 73104 USA; ^8^ Department of Chemistry and University of Missouri Research Reactor University of Missouri Columbia Missouri 65211 United States; ^9^ Institute for Biomedical Engineering Science and Technology (IBEST) Norman Oklahoma 73019 USA

**Keywords:** gold nanoparticles, IGFBP2, IGFBP2/PTEN autoregulation, ovarian cancer, tumor therapy

## Abstract

By exploiting the self‐therapeutic properties of gold nanoparticles (GNPs) a molecular axis that promotes the growth of high‐grade serous ovarian cancer (HGSOC), one of the deadliest gynecologic malignancies with poorly understood underlying molecular mechanisms, has been identified. The biodistribution and toxicity of GNPs administered by intravenous or intraperitoneal injection, both as a single dose or by repeated dosing over two weeks are first assessed; no biochemical or histological toxicity to vital organs is found. Using an orthotopic patient‐derived xenograft (PDX) model of HGSOC, the authors then show that GNP treatment robustly inhibits tumor growth. Investigating the molecular mechanisms underlying the GNP efficacy reveals that GNPs downregulate insulin growth factor binding protein 2 (IGFBP2) by disrupting its autoregulation via the IGFBP2/mTOR/PTEN axis. This mechanism is validated by treating a cell line‐based human xenograft tumor with GNPs and an mTOR dual‐kinase inhibitor (PI‐103), either individually or in combination with GNPs; GNP and PI‐103 combination therapy inhibit ovarian tumor growth similarly to GNPs alone. This report illustrates how the self‐therapeutic properties of GNPs can be exploited as a discovery tool to identify a critical signaling axis responsible for poor prognosis in ovarian cancer and provides an opportunity to interrogate the axis to improve patient outcomes.

## Graphical abstract

Gold nanoparticles inhibit insulin growth factor binding protein 2 (IGFBP2) secretion and expression by disrupting the IGFBP2/mTOR/PTEN axis.

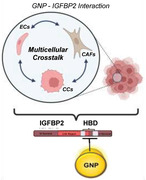

## 1 Introduction

Owing to their tunable optoelectronic, chemical, and biological properties, gold nanoparticles (GNPs) have multiple applications, including as sensory probes, therapeutic agents, drug delivery vehicles, and catalytic agents; GNPs are now under investigation in many preclinical settings.^[^ ^1^, ^2^, ^3^ ^]^ Although generally considered innocuous, with negligible biological activity themselves, increasing evidence suggests that nanoparticles, in fact, possess significant intrinsic biological activity. A variety of nanoparticles of diverse structure, including both organic and inorganic, exhibit therapeutic properties. The self‐therapeutic properties of GNPs have been exploited to inhibit tumor growth, disrupt crosstalk among tumor microenvironmental cells, and as a tool to identify new molecular targets in ovarian and pancreatic cancer, as well as being applied to other conditions including rheumatoid arthritis and eye diseases among others.^[^ ^4^, ^5^ ^]^ We have previously reported the antiangiogenic properties of GNPs; we tested GNPs with diameters of 5, 10, 20, 50, and 100 nm, and showed that those of 20 nm were the most efficacious, and inhibited the function of several heparin‐binding growth factors by altering protein conformations, and thereby suppressing the growth of both ovarian and pancreatic tumors.^[^ ^6^, ^7^, ^8^ ^]^ At the cellular level, the regression of tumor growth was associated with reduced activation of mitogen‐activated protein kinase (MAPK) and reversal of epithelial mesenchymal transition (EMT).^[^ ^6^, ^7^, ^8^ ^]^ We further reported that GNPs transformed activated pancreatic cancer‐associated fibroblasts (CAFs) to quiescence and inhibited the angiogenic phenotype in vitro by disrupting cellular communication between cancer cells and CAFs.^[^ ^9^, ^10^ ^]^ We have also demonstrated the utility of GNPs as a tool to capture proteins of interest.^[^ ^11^, ^12^ ^]^ Upon interacting with a biological system, GNPs rapidly adsorb various molecules to form a protein corona on the surface; this protein corona significantly impacts the biological properties of the particle. We exploited the protein corona modulation around GNPs and identified several new targets (i.e., HDGF, SMNDC1, PPA1, PI15, gasdermin B, and IGFs) in ovarian cancer.^[^ ^11^ ^]^ In toto, this evidence suggests that GNPs can serve as a unique tool to interrogate and identify critical molecular axes responsible for disease progression. Herein, we confirm this application of GNPs and utilize it successfully to identify a critical signaling axis that promotes ovarian cancer growth.

## 2 Result and Discussion

### 2.1 Optimization of Dose and Route of Administration for Gold Nanoparticles Through the Assessment of Toxicities In Vivo

**Figure 1:**
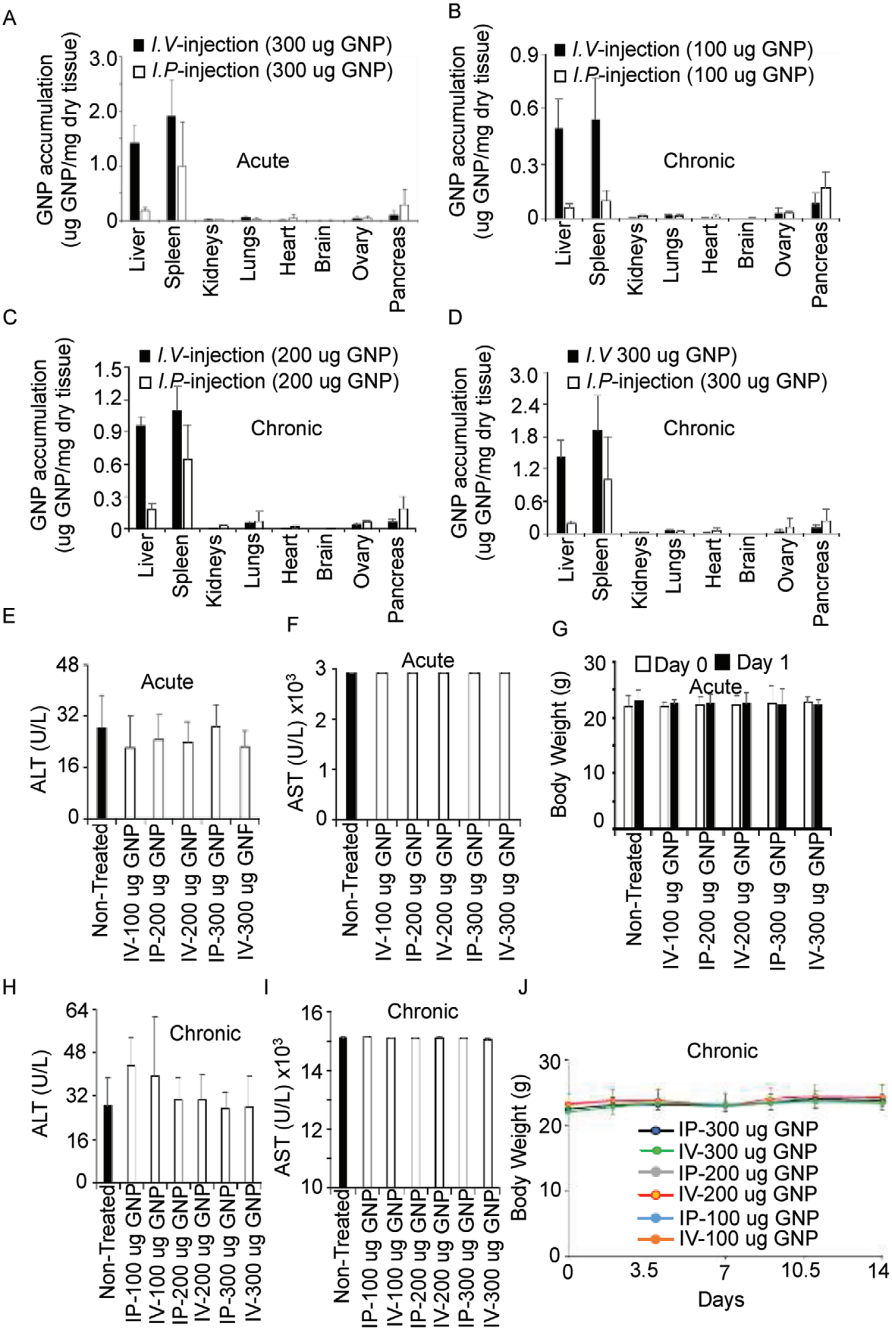
Bioaccumulation of gold nanoparticles did not induce toxicities. A) Single dose accumulation. Mice (*n* = 5) were intravenously or intraperitoneally injected with a single dose (300 µg) of GNPs (acute dosing). After 24 h, liver, spleen, kidneys, lungs, heart, brain, ovary, and pancreas were processed to quantify GNP content using INAA. Accumulation is shown as µg GNPs/mg dry tissue. B–D) Multiple dose accumulation. Mice were intravenously or intraperitoneally injected with various doses (100, 200, and 300 µg) of GNPs every other day for 14 days (chronic dosing). The liver, spleen, kidneys, lungs, heart, brain, ovary, and pancreas were processed to quantify GNP content using INAA. Accumulation is shown as µg GNPs/mg dry tissue. E,F) Plasma were collected from mice receiving single doses (acute dosing) and ALT and AST were measured by colorimetric analysis as a measure of liver toxicity. G) Body weights of mice receiving single doses were assessed twice in 24 h. H,I) Plasma was collected from mice receiving multiple doses (chronic dosing) and ALT and AST were measured by colorimetric analysis as a measure of liver toxicity. J) The body weights of mice receiving multiple doses were assessed for 14 days.

**Figure 2:**
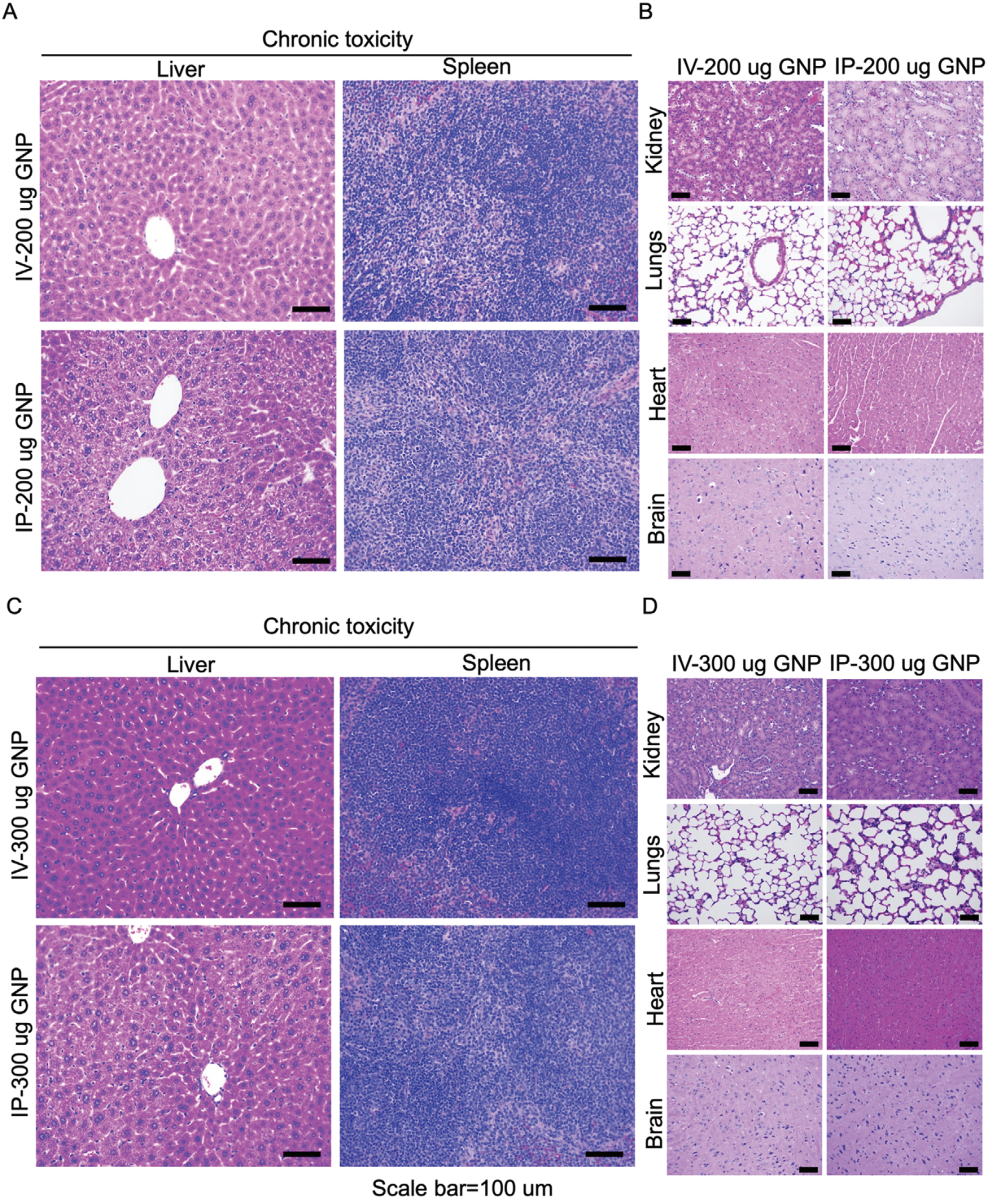
Histopathological analysis for the evaluation of toxicities induced by gold nanoparticles. A–D) Histopathological analysis. H&E‐stained tissue sections from mice receiving multiple doses of 200 or 300 µg of GNPs given with intravenously or intraperitoneally (chronic dosing). Images of liver and spleen sections (A,C) and kidneys, lungs, heart, and brain (B,D) were captured by a Nikon microscope. Scale bar, 100 µm.

The bioavailability of any therapeutic is largely determined by the dose and route of administration, both of which can be manipulated to maximize therapeutic efficacy and minimize toxicity.^[^ ^13^ ^]^ Since we previously reported that GNPs of 20 nm size demonstrated the highest biological activity,^[^ ^6^, ^7^, ^8^ ^]^ therefore, in the current study we synthesized and used GNPs of ≈20 nm in size. GNPs were synthesized using the citrate reduction method, as previously described,^[^ ^9^ ^]^ and were physicochemically characterized by dynamic light scattering (DLS), zeta potential measurements, and Transmission Electron Microscopy (TEM). The size and surface charge of the GNPs were determined by DLS and zeta potential measurements respectively. DLS showed the GNPs had a diameter of ≈21 nm, while zeta potential measurements showed a net negative charge of ≈−44 mV (Figure S1A‐B, Supporting Information). TEM confirmed the morphology of GNPs, and revealed a spherical shape (Figure S1C). To optimize the dose and route of administration of GNPs, we compared two routes of administration that are commonly used in the treatment of ovarian cancer, i.e., the intravenous (*i.v*.) and intraperitoneal (*i.p*.) routes. We also compared a range of GNP doses, given either as a single dose or as multiple doses over a period of two weeks. Since the bioaccumulation of GNPs may be associated with toxicities in vivo, we first wanted to determine the biodistribution of injected GNPs in various body tissues to assess both dose‐related toxicities and the effect of the route of administration. Normal mice received GNPs by either *i.v*. or *i.p*. injection (300 µg per mice) and the bioaccumulation of gold in various organs, specifically liver, spleen, kidney, lungs, heart, brain, ovary, and pancreas was measured by instrumental neutron activation analysis (INAA). Twenty‐four hours after administration (Acute setting), GNPs were found predominantly in highly metabolic organs, i.e., the liver and spleen; GNP levels in metabolic organs were slightly lower following *i.p*. administration compared to *i.v*. administration, while accumulation in nonmetabolic organs was similar in both routes. Bioavailability of intravenously administered drugs in metabolic organs shows higher accumulation than *i.p*. administration (**Figure** **1**A). We next quantified GNP accumulation following repeated administration of GNPs, in order to adequately reflect therapeutic reality. Mice were administered GNPs at various doses (100, 200, and 300 µg) by the *i.v*. or *i.p*. routes every 2 days for 14 days (Chronic setting). GNP accumulation was similar to that seen with the single dose, although the accumulation of GNPs increased in a dose‐dependent manner (Figure 1B–D). Next, we investigated whether these high accumulations in the liver were toxic; we assessed liver function by measuring blood levels of the liver enzymes aspartate aminotransferase (AST) and alanine aminotransferase (ALT); levels of these enzymes are elevated in blood following liver damage.^[^ ^14^, ^15^ ^]^ Levels of AST and ALT in serum were similar in both treated and non‐treated mice, indicating that GNPs did not induce liver damage at either 1‐day (acute) or 14‐days (chronic), despite ≈20% of the GNPs accumulating in the liver following *i.v*. injection (Figure 1E–H). In addition, no significant change in body weight was seen at any GNP dose (Figure 1I–J). Lack of toxicity was also confirmed by histochemical analysis of tissues collected from the liver, spleen, kidney, lungs, heart, and brain of treated mice (**Figure** **2**). In summary, we did not detect either any acute or chronic toxicity with GNPs in this study (Figures 1 and 2). Several other studies also report that both negative and neutral GNPs mainly accumulate in the liver irrespective of size and route of administration, and may induce liver damage.^[^ ^16^, ^17^ ^]^ The fact that we did not find any toxicity associated with ≈20 nm citrate‐capped GNPs, may reflect their synthesis with citrate and their negative‐charge consistent with another report.^[^ ^18^ ^]^ Collectively, this study confirms that 20 nm citrate‐capped negatively charged GNPs are nontoxic, following either single‐ or multi‐dose administration to mice, irrespective of dose or route of administration.

### 2.2 Gold Nanoparticles Inhibit Orthotopic Tumors in a Murine PDX Model

**Figure 3:**
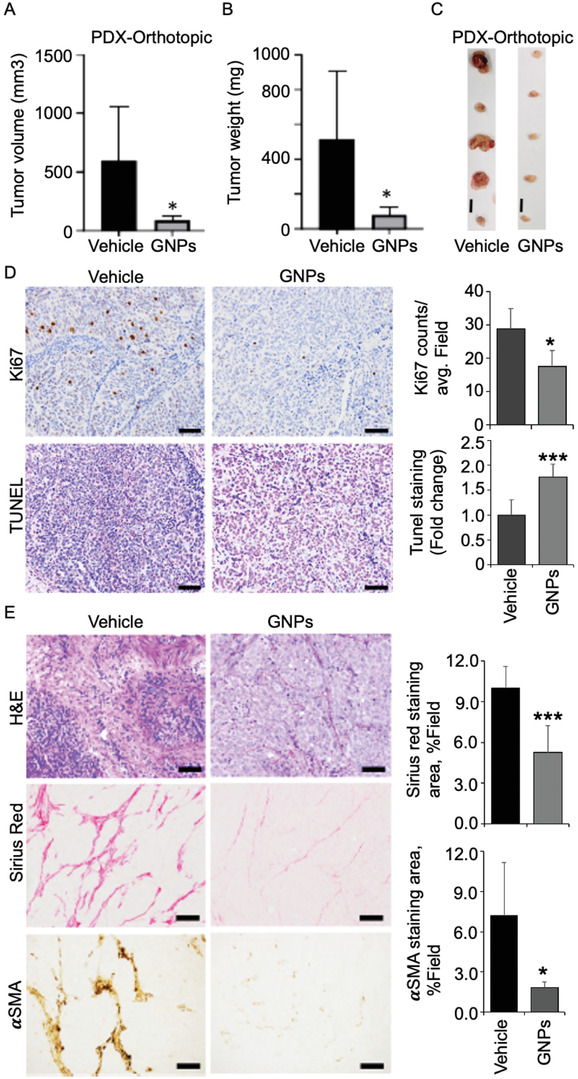
Gold nanoparticles inhibit orthotopic tumors in a murine PDX model. A–C) Assessment of antitumor efficacy of GNPs in an orthotopic PDX model mice. PDX‐098 was orthotopically transplanted into the ovary of NOD/SCID background mice (*n* = 5). Tumor‐bearing mice (tumor size ≈100 mm^3^) were intravenously injected with GNPs (10 mg kg^−1^ per thrice weekly) or vehicle (PBS) for 21 days. The tumor volume (A) and tumor mass (B) were measured, and tumor images (C) were captured at 21‐day. D) Representative Ki67‐stained (upper) and TUNEL‐stained (bottom) sections of PDX tumor. The quantification of Ki67‐stained proliferating cells (top), and TUNEL‐stained apoptotic cells (bottom) (*n* = 15 from five mice). E) H&E, *α*‐SMA, and Sirius red‐stained sections. The quantification of Sirius red‐stained cells (top), and *α*‐SMA‐stained cells (bottom) (*n* = 15 from five mice). The intensities of images were quantified using ImageJ and analyses were performed using a student *t*‐test. *P ≤ 0.05, **P ≤ 0.01, and ***P ≤ 0.001. *n* = 15 (D,E). Statistical analyses were performed using one‐way ANOVA followed by Dunnett's multiple comparisons test. *P ≤ 0.05, *n* = 5 (A,B). Data are expressed as means ± SD.

**Figure 4:**
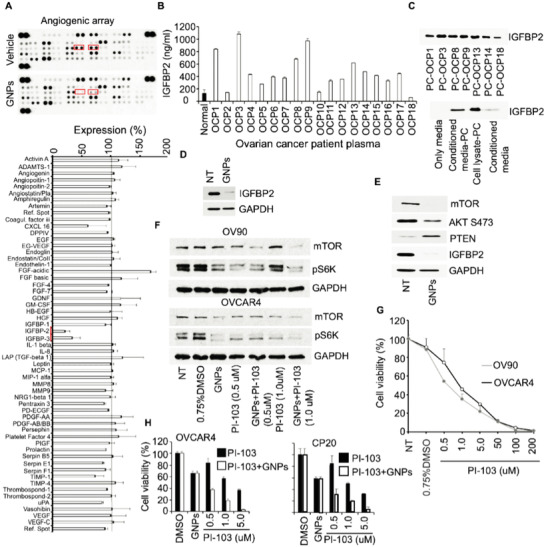
Gold nanoparticles provide anti‐tumor activity through an autoregulatory feedback loop of IGFBP2/PTEN interaction. A) Identification of GNP‐altered angiogenesis‐related proteins in plasma. Typical images of the antibody arrays incubated with plasma pool of GNP‐ and vehicle‐treated mice (A upper panel). Lower panel shows the pixel density ratio of GNP versus vehicle (*n* = 3); IGFBP2 and IGFBP3 are highlighted with red boxes in both the upper and lower panels. B) Detection of insulin growth factor binding protein‐2 (IGFBP2) in the plasma of ovarian cancer patients. IGFBP2 levels in plasma from ovarian cancer patients (*n* = 18; designated OCP1‐OCP18) were measured by ELISA. C) Detection of IGFBP2 bound to GNPs via formation of the protein corona. Plasma from seven patients (OCP1, OCP3, OCP8, OCP9, OCP13, OCP14, and OCP18) were incubated with 50 µg GNPs for 18 h and the protein corona (PC; designated PC‐OCP1, PC‐OCP3, PC‐OCP8, PC‐OCP9, PC‐OCP13, PC‐OCP14, and PC‐OCP18) analyzed by immunoblotting (upper panel). Ovarian cancer cells (OV90) were cultured overnight in complete media; media was then replaced with conditioned media (no FBS media) and 48 h later both the conditioned media and cell lysates were incubated with 50 µg GNPs for 18 h and the formed PCs (media‐PC and cell lysate‐PC) analyzed by immunoblotting (lower panel). D) Suppression of IGFBP2 by GNPs. After 24 h of regular culture, OV90 cells were treated with GNPs (25 µg ml^−1^) in the presence of conditioned media. 48 h later, IGFBP2 was determined by western blotting (WB). E) Downregulated IGFBP2 by GNPs follows PTEN‐mediated signaling pathway. OV90 cells were treated with GNPs (25 µg ml^−1^) or remained untreated for 48 h and then the expression of mTOR, PTEN, AKT, and IGFBP2 in lysates was determined by WB. GAPDH was used as the loading control. F) PI‐103 deactivates kinase activities. Ovarian cancer cells (OV90 and OVCAR4) were treated with DMSO, GNPs (25 µg ml^−1^) or PI‐103 at various doses (0.5, 1.0, and 5.0 µM). After 48 h, the expression of mTOR and S6K in lysates was determined by WB. GAPDH was used as the loading control. G) Dose optimization for cell viability. Ovarian cancer cells (OV90 and OVCAR4) were treated with PI‐103 at various doses (0.5, 1.0, 5.0, 50, 100, and 200 µM), with DMSO, or were not treated (NT). After 48 h, cell viability was assessed by counting the cells using a hemocytometer. H) Assessment of cell growth inhibition by the combined delivery of GNPs and PI‐103. Ovarian cancer cells (OVCAR4 and CP20) were either treated with PI‐103 at various doses (0.5, 1.0, 5.0 µM) or pretreated with GNPs and then treated with the same doses of PI‐103. DMSO and GNPs (25 µg ml^−1^) served as their corresponding controls. After 48 h, cell viability was assessed by counting the cells using a hemocytometer.

Since no obvious toxic effects were seen in vivo with our 20 nm GNPs, we next examined their antitumor efficacy against an ovarian patient‐derived xenograft (PDX) model in immunodeficient nonobese diabetic/severe compromised immunodeficiency (NOD/SCID) mice. Before transplantation of ovarian patient‐derived tumor tissue, we first assessed the tumorigenic properties of six patient‐derived ovarian cancer tissues, by determining expression levels of alpha‐smooth muscle actin (*α*‐SMA) and the endothelial cell marker CD31 by immunohistopathology. Expression of *α*‐SMA and CD31 was very high in the PDX‐098 tissues, indicating activated tumor microenvironment (Figure S2, Supporting Information). In ovarian cancer, expression levels of *α*‐SMA and CD31 are highly correlated with the malignant potential of ovarian tumors.^[^ ^5^, ^19^ ^]^ Having determined their malignant potential, we next transplanted patient‐derived ovarian tissues orthotopically in NOD/SCID female mice (20 mice). When tumor became palpable, we divided the mice into two groups of 10 mice; one group received *i.v*. injection of GNPs (200 µg three times weekly), the second received vehicle (PBS) only, and served as a control group. The treatment was continued for 21 days (9 GNP injections in total), at which point mice were euthanized and tumors were collected, weighed, and photographed (Figure A‐C). Treatment with GNPs significantly inhibited tumor growth compared to PBS treatment. Inhibition of tumor growth was also shown by staining for a decrease in proliferating cells (Ki67 staining) and an increase in apoptotic cells [TUNEL (terminal deoxynucleotidyl transferase‐mediated deoxyuridine triphosphate nick end labeling) staining] (**Figure** **3**D). Histological morphology of the tumor tissue as revealed by hematoxylin and eosin (H&E) staining exhibited lower densities of tumor cells and increased pyknotic nuclei in the GNP‐treated group compared to the vehicle group (Figure 3E). A decrease in Sirius Red staining and *α*‐SMA demonstrated a reduction of the amount of fibrosis/collagen fibers (such as activated fibroblast‐like cells and collagen types I and III) (Figure 3E). For neovascularization, pericyte/endothelial cell interactions are crucial,^[^ ^20^, ^21^, ^22^ ^]^ and abnormalities in such cell interactions lead to defects in vessel morphogenesis, maturation, and function.^[^ ^20^, ^22^, ^23^, ^24^ ^]^ Therefore, we immuno‐stained tumor tissues using pericyte (NG2) and endothelial cell (CD31) markers. Our results suggest that GNPs can also prevent the cross‐talk between pericytes and endothelial cells, as evidenced by a decrease in colocalization of CD31 and NG2 stain, and thus halt tumor growth (Figure S3, Supporting Information). We have also visualized the distribution of gold nanoparticles in tumor tissues of PDX model by confocal laser scanning microscopy (CLSM). It is evident from the CLSM study that GNPs were distributed in the tumor tissues of PDX tumors (Figure 4SA, Supporting Information). In total, these data show that 20 nm GNPs represent an effective therapy leading to regression of the ovarian tumor in PDX model mice.

### 2.3 Gold Nanoparticle Showed Anti‐Tumor Activity Through an Autoregulatory Feedback Loop of IGFBP2/PTEN Interaction

We next sought to define the molecular and cellular mechanisms mediating the antitumor activity of the GNPs. The decrease in Ki67, CD31, and *α*‐SMA staining described above suggests that GNP treatment is disrupting crosstalk among tumor microenvironmental cells, possibly by regulating the secretion of cell‐communicating factors such as growth factors and cytokines. Therefore, we focused on alterations in angiogenesis‐related proteins in the plasma of PDX tumor‐model mice that were treated either with GNPs or vehicle. We performed an antibody array‐based immunoblot assay which could detect the expression of 55 proteins associated with angiogenesis and tumor progression. The intensity and size of spots on the resulting dot blots reflect the expression of each detected protein in plasma (**Figure** **4**A, upper panel). To quantify protein expression, the pixel density of each spot was analyzed using ImageJ, and the ratios for GNP‐treated mice to vehicle‐treated control mice, were calculated. Among the 55 proteins, the plasma levels of IGFBP2 (insulin growth factor binding protein 2) and IGFBP3 (Insulin Growth Factor Binding Protein 3) were downregulated at ≈80% and 50%, respectively, by GNP treatment, whereas the expression of FGF acidic was significantly upregulated (Figure 4A, lower panel). These data suggested that GNPs alter the plasma level of IGF‐related proteins in PDX mice and that IGFBP2 may play a critical role in ovarian cancer growth. Since IGFBP2 was more robustly downregulated than IGFBP3, we next sought to investigate whether IGFBP2 secretion is increased in ovarian cancer. To assess a potential role of IGFBP2 in ovarian cancer we determined the plasma level of IGFBP2 in 18 ovarian cancer patients using ELISA. Elevated IGFBP2 was detected in 83% of ovarian cancer patient plasmas, suggesting that IGFBP2 expression positively correlated with malignant progression (Figure 4B), consistent with other reports.^[^ ^25^, ^26^, ^27^ ^]^ Importantly, increasing evidence also indicates that IGFBP2 is positively associated with the malignant progression of numerous other cancers such as glioma,^[^ ^28^ ^]^ as well as breast, prostate,^[^ ^29^ ^]^ lung,^[^ ^30^ ^]^ colon^[^ ^31^ ^]^ and lymphoid^[^ ^32^ ^]^ cancers.

IGFBP2 is a heparin‐binding (HB) domain‐containing protein, and we previously reported that GNPs bind to HB‐domain containing proteins via the HB domain and in the process alters protein conformation and thereby inhibiting protein function. Thus, we next investigated whether GNPs can sequester IGFBP2 from human patient plasma, cancer cell lysates, or cancer cell‐conditioned media (i.e., secreted into growth media by ovarian cancer cells under starving conditions) as a possible mechanism to inhibit IGFBP2 function and thus tumor cell growth. Immunoblotting of the GNP sequestered protein from patient plasma confirmed the presence of IGFBP2, and thus the ability of GNPs to sequester IGFBP2 from the patient plasma. (Figure 4C, upper panel). Similarly, immunoblotting of the GNP sequestered protein revealed that when GNPs were incubated with either cell lysate or conditioned media, the protein corona contained high levels of IGFBP2 that were significantly enriched compared to the original cell lysates and conditioned media. (Figure 4C, lower panel). Protein corona formation ≈20 nm GNPs was confirmed by DLS, Zeta potential, UV‐visible spectroscopy, BCA assay, and gel electrophoresis (Figure S5, Supporting Information). DLS measurements revealed that as‐synthesized GNPs had a hydrodynamic diameter of ≈26 nm that increased to ≈46 nm following incubation with lysate proteins (Figure S5A,C, Supporting Information). Similarly, the charge of as‐synthesized GNPs changed from ≈ ‐49 mV to ≈ ‐28 mV, indicating protein adsorption to the GNP surface (≈Figure S5B,D, Supporting Information). Furthermore, increased absorbance of GNPs following incubation with the lysate, and its stabilization by 10% NaCl, confirms protein binding to GNPs (Figure S5E,F, Supporting Information). Finally, quantitation of protein in the protein corona by BCA assay revealed ≈ 43 µg of bound protein (≈Figure S5G, Supporting Information). Collectively, these results demonstrate the formation of a protein corona around GNPs and that the GNPs sequester IGFBP2, indicating that at the molecular level, the tumor regression reported in the PDX model following GNP treatment may be associated with a decrease in IGFBP2 plasma levels.

Next, we wanted to investigate whether the decrease in IGFBP2 levels in plasma could be associated with the downregulation of cellular IGFBP2 expression upon GNP treatment. Treatment of ovarian cancer cells with GNPs decreased expression of IGFBP2, compared to untreated cells (Figure 4D). It is reported that cellular expression of IGFBP2 is orchestrated by an autoregulatory feedback loop via the IGFBP2/mTOR/PTEN axis. The binding of IGFBP2 to cell surface integrin receptors decreases PTEN expression, which in turn, activates Akt and mTOR signaling to increase IGFBP2 transcription and hence protein translation.^[^ ^33^, ^34^, ^35^, ^36^ ^]^ Therefore, we wanted to determine whether treatment with GNPs alters the expression of mTOR and PTEN in ovarian cancer cells. Treatment with GNP robustly downregulated expression of IGFBP2, mTOR as well as activation of AKT (p‐S473), while increasing expression of PTEN, suggesting disruption of IGFBP2 autoregulation via mTOR/PTEN axis (Figure 4E). This finding suggests that the opposite would hold during tumor progression, i.e., the PI3K/AKT/mTOR pathway is activated by IGFBP2 while PTEN is deactivated; this phenomenon is common across a variety of human cancers as reported by others.^[^ ^33^ ^]^ Previously, Wang et al. reported that dietary GNPs can marginally activate the PI3K/Akt/mTOR pathway and lipid metabolism in Drosophila larvae.^[^ ^37^ ^]^ However, in‐depth molecular mechanism of this observation still needs to be elucidated. In contrast to the inhibitory action of decreased IGFBP2 mediated by GNPs on PI3K/AKT/mTOR pathway as we presented here, the activation of the pathway by dietary GNPs may be, in part, due to i) contribution due to the interaction of GNPs with diet components; ii) aberrant activation of the pathway in cancer versus normal physiology and; iii) a different method of preparation and low concentration of GNPs used.

Thus, our conclusion is that the decreased IGFBP2 expression caused by GNP treatment deactivates the PI3K/Akt/mTOR signaling pathway, while activating PTEN, via dysregulation of an autoregulatory feedback loop involving IGFBP2/PTEN interaction.

Since the AKT/mTOR pathway was deactivated by GNPs, we next interrogated whether inhibition of the AKT/mTOR pathway is a mechanism of the anti‐tumor activity of GNPs and whether inhibition of AKT/mTOR could be exploited as a therapeutic approach in ovarian cancer. We additionally examined whether the antitumor activity of GNPs would be enhanced by the simultaneous administration of a new potent PI3K/Akt and mTOR inhibitor (PI‐103). We initially sought to optimize the dose of PI‐103; first, we assessed the impact of PI‐103 on the mTOR/S6K pathway which is involved in growth signaling.^[^ ^38^, ^39^ ^]^ Ovarian cancer cells were treated with PI‐103 at one of two doses, either with or without GNPs. The data show that PI‐103 inhibits the mTOR/S6K growth signaling pathway in a dose‐dependent manner, and that the impact is enhanced by combined treatment with GNPs (Figure 4F). Second, we assessed whether inhibition of mTOR signaling is reflected in ovarian cancer cell viability. To test the impact of mTOR signaling inhibition on cellular viability, ovarian cancer cells were treated with PI‐103 at various concentrations and cell viability was determined; we found that PI‐103 inhibited ovarian cancer cell growth in a dose‐dependent manner (Figure 4G). We then assessed the impact of combined PI‐103 and GNP treatment on cell viability using concentrations of PI‐103 that reduced cell viability by ≈20%–70% when given alone. The combination of GNPs and PI‐103 significantly enhanced the growth inhibition of ovarian cancer cells, compared to PI‐103 alone (Figure 4H). These results suggest that aside from their effect on the IGFBP2/mTOR/PTEN axis, GNPs may also inhibit other pathways to inhibit ovarian cancer cell growth and viability, at least in *vitro*.

### 2.4 Gold Nanoparticles in Combination with Dual Kinase Inhibitor PI‐103 Exhibits Potential Inhibition of Tumors in a Human Xenograft of Ovarian Cancer

**Figure 5:**
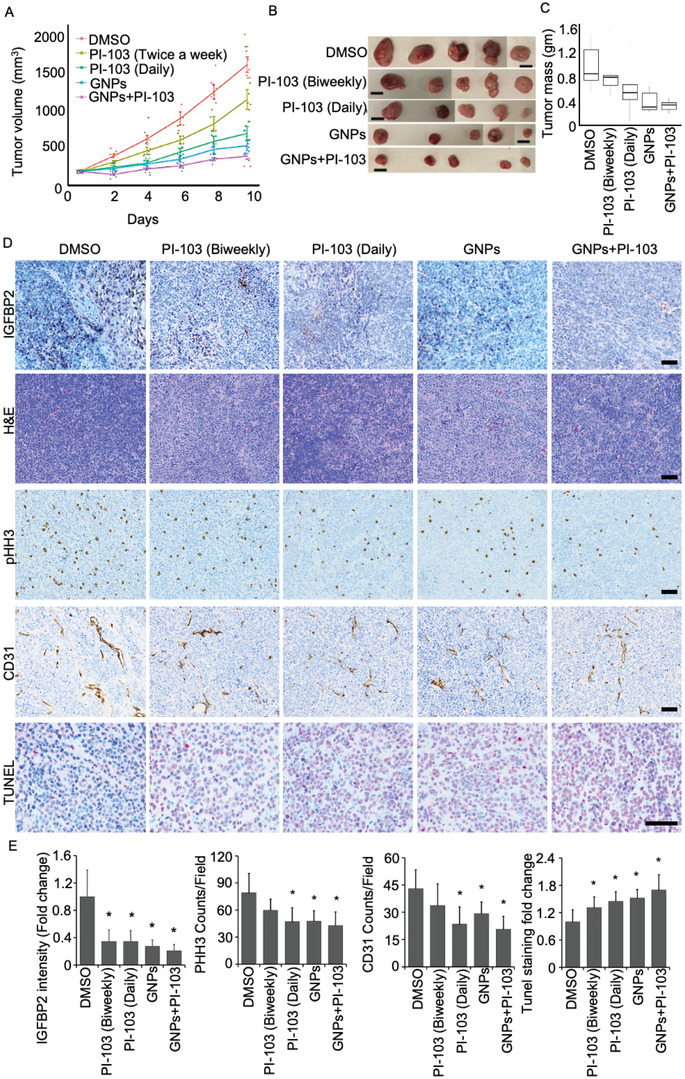
Gold nanoparticles in combination with dual kinase inhibitor PI‐103 exhibit inhibition of tumors in a human xenograft of ovarian cancer. A–C) Reduction in tumor growth by combined delivery of GNPs and PI‐103. Tumor harboring mice were treated with DMSO and with intravenous injections of GNPs, PI‐103 (daily or twice weekly, intraperitoneally), or GNPs plus PI‐103 for 10‐days. Tumor volume (A), tumor images (B), and tumor mass (C) at 10 days are shown. Data are expressed as means ± SD and one‐way analysis of variance (ANOVA) followed by Dunnett's multiple comparisons test. *P ≤ 0.05, **P ≤ 0.01, ***P ≤ 0.001, and ****P ≤ 0.001, *n* = 8. D) Tumor tissues after completion of Day10 treatments, 8 mice from each group were taken out for IHC staining of tumor xenografts for IGFBP2, pHHB3, TUNEL, CD31 and H&E staining. Scale bar represents 100 µm. E) Quantification of intensity. Slides were immunostained with IGFBP2, pHHB3, TUNEL, and CD31. Positive cells or intensities were counted using ImageJ. The fold changes or counts/filed with respect to the control are graphically shown. Control is DMSO treated control; PI‐103 (twice weekly); PI‐103 (daily); GNPs (thrice weekly) and GNPs (thrice weekly) +PI‐103 (twice weekly). *P < 0.05 compared with the control group by student *t*‐test.

Having established the efficacy of the PI‐103/GNP combination in vitro, we evaluated the therapeutic response in a human xenograft model of ovarian cancer by implanting OV90 cells subcutaneously in athymic nude mice. When tumors reached ≈175 mm^3^, mice were randomly assigned to one of five groups (*n* = 8 per group); treatments were PI‐103 alone (either daily or twice weekly), GNPs alone, GNPs combined with PI‐103 (twice weekly), or dimethyl sulfoxide (DMSO) as control. GNPs were administered intravenously three times a week (10 mg kg^−1^–200 µg per animal), PI‐103 was administered intraperitoneally (5 mg kg^−1^ on either schedule); for the combined therapy group GNPs (10 mg kg^−1^) were given three times a week and PI‐103 (5 mg kg^−1^) twice a week. Animals were monitored for distress daily, and tumor size was measured every other day (**Figure** **5**A). All animals were euthanized at completion, tumors were excised and tumor size and weight were determined (Figure 5B,C). We used the linear mixed model to analyze the tumor volume change across 10 days by the five groups. There is a significant interaction between groups and time in days. Comparing to DMSO group, PI‐103 (twice weekly) group was estimated to have a reduced tumor volume of 50.14 every day *(P* < .001). All other group comparisons of tumor volume change per day are shown in Table S1, Supporting Information. Using multiple testing corrected significant threshold of 0.005 (0.05/10), we found no significant tumor change per day between the GNP and daily PI‐103 groups, or between the GNP and the PI‐103/GNP combination groups. All other group comparisons were significant. We used the Kruskal‐Wallis test to compare tumor weights at the end point among the five groups. There was a significant difference in tumor weight across the five groups (*P* < .001). We also used Wilcoxon rank sum exact test to make pairwise comparisons of tumor weight difference between pairwise groups. Using multiple testing corrected significant threshold of 0.005 (0.05/10), we found significant end point tumor weight differences for the following group comparisons: the GNP and DMSO groups, the GNP and twice weekly PI‐103 groups, the DMSO and PI‐103/GNP combination groups, and the twice weekly PI‐103 and PI‐103/GNP combination groups. No other group comparisons were significant; results are summarized in Table S2, Supporting Information.

In order to better understand and molecularly characterize the antitumor efficacy of GNPs and/or PI‐103 we used immunohistochemistry (IHC) to assess the expression of IGFBP2, phosphohistone H3 (pHHB3) as a proliferation marker (mitosis), CD31 as an endothelial marker, and TUNEL positive nuclei as an apoptosis marker. Upon completion of treatment, we determined expression of IGFBP2, pHHB3, CD31, and TUNEL; expression of IGFBP2 was significantly reduced in both the GNP‐treated group and in the GNP/PI‐103 combination group (Figure 5D, upper panel). Similar reductions in IGFBP2 expression were seen in the PI‐103 treated group. Expression of the mitotic tumor cell proliferation marker (pHHB3) was also significantly reduced in all treatment groups compared to the DMSO control (Figure 5D). The GNP/PI‐103 combination group showed a significant increase in TUNEL positive nuclei (1.7‐fold), compared with the DMSO control, suggesting enhanced apoptosis (Figure 5D, lower panel). In addition, there was a significant decrease in the CD31 positive microvessel density in the GNP (1.4‐fold) and GNP/PI‐103 combination (2.1‐fold) groups, compared with the DMSO control, demonstrating inhibition of angiogenesis (Figure 5D). These results show that the GNP/PI‐103 combination reduces the expression of IGFBP2 at the tissue level in vivo and that the antitumor efficacy is mediated by an increase in apoptotic death. Furthermore, H&E staining of tumor tissues showed reduced densities of tumor cells in all the treatment groups compared to the DMSO control (Figure 5D). In addition, we also observed that GNPs were distributed in tumor and liver tissues in these animals (Figure 4SB, Supporting Information).

The PI3K‐AKT‐mTOR pathway is one of the main signaling node by which cancer cells survive and proliferate; thereby it is at the center of anti‐cancer drug development.^[^ ^40^, ^41^ ^]^ Unfortunately, targeted inhibition of a single kinase is fraught with limited therapeutic benefit, mainly due to the evolution of drug resistance.^[^ ^42^ ^]^ This limitation could be overcome, in part, by inhibiting multiple pathological pathways using combination therapy. As an example, the use of BET bromodomain inhibitor in combination with PARP inhibitor synergistically increased DNA damage and cell‐cycle checkpoint defects, causing a mitotic catastrophe in BRCA‐proficient ovarian cancer.^[^ ^43^ ^]^ In addition, the combination of PI3K inhibitor and BET protein inhibition provided therapeutic benefit in metastatic breast cancer.^[^ ^44^ ^]^ Importantly, we previously reported that GNPs sensitize pancreatic cancer (PC) cells to gemcitabine by reversing epithelial to mesenchymal transition (EMT), reducing cancer stemness, and inhibiting mitogen activated protein kinase (MAPK) activation.^[^ ^8^ ^]^ It is generally recognized that monotherapies have minimal effects on survival in cancer patients and combination therapies are usually recommended. Therefore, in our present study, we also wanted to check if combining GNPs with PI‐103 could enhance the therapeutic efficacy. Although the in vitro viability data suggest an enhanced therapeutic effect from the GNP/PI‐103 combination treatment, the in vivo data did not reveal a statistically significant difference in inhibition of tumor growth between the GNP and the combination group. Thus, it is likely that, in addition to the IGFBP2/mTOR/PTEN axis, GNPs interfere with other signaling pathways, at least in vitro. Furthermore, escalating doses of drugs are fraught with dose‐limiting toxicities. Therefore, we sought to test if a combination treatment could improve therapeutic efficacy and reduce systemic toxicity. Specifically, we assessed if combination therapy of GNPs and PI‐103 could inhibit ovarian tumor growth. Taken together, these results indicate that combination treatment may improve the therapeutic efficacy in ovarian cancer, at least in vitro.

In summary, we have identified a new regulatory protein, IGFBP2, by which GNPs operate to impair the development and progression of ovarian cancer. On the basis of bioaccumulated GNPs, we first determined a non‐toxic dose of GNPs and used that dose to demonstrate inhibition of tumor growth in an orthotopic PDX model mouse. The antitumor activity was mediated by an autoregulatory feedback loop of IGFBP2/PTEN interaction through deactivation of the PI3K/Akt/mTOR growth signaling pathway and activation of the survival protein PTEN. In addition, antitumor activity was similar to combining GNP therapy with a low dose of a small molecule dual phosphokinase inhibitor (PI‐103). This study demonstrates a novel application of self‐therapeutic nanoparticles as a tool to identify key signaling axes responsible for tumor growth. As a proof‐of‐concept study, we applied GNPs to identify and validate a promising target protein, IGFBP2, responsible for the malignant progression of ovarian cancer and showed that the anti‐tumor efficacy of GNPs is, at least in part, mediated by the reduction of IGFBP2 levels. GNPs thus represents a promising therapy for ovarian cancer, either alone or in a drug combination (e.g., with PI‐103); such a therapy would be extremely valuable given the currently limited treatment options and generally poor outcomes for ovarian cancer patients and could relatively quickly translate to the clinic.

## 3 Experimental Section

### Materials

Gold (III) chloride trihydrate (520 918) and sodium citrate tribasic trihydrate (S4641) were bought from Sigma‐Aldrich (St. Louis, MO). Cell culture media RPMI 1640 (10‐040‐CV) was obtained from Corning Inc. (Corning, NY, USA). FBS (16000‐044) and Penn‐Strep (15140‐122) were purchased from Life Technologies (Grand Island, NY, USA), Opti‐MEM was from Thermo Fisher Scientific (Waltham, MA, USA). The following primary antibodies were purchased from the specified vendor: rabbit monoclonal anti‐IGFBP2 (#3922S), ‐PTEN (#9552S), ‐mTOR (#2972S) and ‐AKT S473 (#4058S) (Cell Signaling Technology, Danvers, MA, USA), rabbit anti‐GAPDH (#9545) and ‐tubulin (#T5168) (Sigma Aldrich). Lipofectamine RNAiMAX transfection reagent (13 778 150) was bought from Invitrogen (Waltham, Massachusetts).

### Synthesis and Characterization of 20 nm Gold Nanoparticles

20 nm GNPs were prepared as we previously described.^[^ ^7^, ^8^, ^9^ ^]^ Briefly, 10 mM gold III chloride trihydrate solution (cat. 520 918, Sigma‐Aldrich, St. Louis, MO, USA) (5 ml) was diluted 40 times with endotoxin‐free water and heated to boiling. Prewarmed 1% sodium citrate tribasic trihydrate solution was added to a final volume of 200 ml and the solution was maintained at boiling for 10–15 min until the solution become dark purple. The solution was cooled to room temperature and stirred overnight. Synthesized GNPs were characterized by dynamic light scattering (DLS), zeta potential measurement (Malvern Zetasizer Nano ZS), and TEM microscopy as previously described.^[^ ^6^, ^9^, ^11^ ^]^ For the measurement of size and zeta potential, 50 µg ml^−1^ as synthesized gold nanoparticle is used, where the ionic strength of Tetrachloroauric (III) acid trihydrate (HAuCl_4_.3H_2_O) and trisodium citrate dihydrate (HOC(COONa)(CH_2_COONa)_2_.2H_2_O) solution are 0.1 mol L^−1^ and 0.272 mol L^−1^ in deionized water, respectively.

### Qualitative and Quantitative Observation of Accumulated Gold Nanoparticles in Various Organs

For the quantification of accumulated GNPs in various organs of mice, after sacrifice, tissues from the liver, spleen, kidneys, lungs, heart, brain, ovary, and pancreas of each mouse were collected and analyzed by INAA as previously described.^[^ ^45^ ^]^ Briefly, each collected tissue was weighed and transferred into a precleaned, high‐density polyethylene irradiation vial and was then lyophilized to constant dry weight. Dry tissue was reconstituted with 100 µL sample solution, loaded in polyethylene transfer “rabbits” and irradiated for 90 s in a thermal flux density of ≈5×10^13^ ncm^−2^ s^−1^. The reconstituted sample was then allowed to decay for 24–48 h and counted in real‐time on a high‐purity germanium detector for 3600 s at a sample‐to‐detector distance of ≈5 cm. Gold mass was quantified by measuring the 411.8 keV gamma ray from *β*‐ decay of 198Au (t1/2 = 2.7 days), and calibrated using certified gold standard solutions.

For the observation of accumulated GNPs into mice, after eu euthanizati, the GNPs‐and controls‐treated tumor and liver tissues were fixed in 10% formalin, embedded in paraffin, and sectioned (4 µm) using a Leica multistainer (ST5020) following standard protocols. The mounted tissue slices of livers and tumors were imaged using a Zeiss LSM 880 confocal laser scanning microscope on the Zeiss Zen Black software using a 63X oil objective (NA = 1.4) with photomultiplier tube (PMT) detectors. H&E stains were visualized through the transmitted light of a 405 nm diode laser through a main beam splitter (MBS) 488/561/633 filter to the PMT detector (T‐PMT). The AuNPs were imaged using light scattering as previously described^[^ ^46^, ^47^, ^48^ ^]^ with a 633 nm helium‐neon laser and a MBS T80/R20 filter, using a detector range of 633 nm +/− 10 nm. Following image acquisition, the images were then processed using the Zeiss Zen Lite software to threshold the light scattering signals to remove the background light scattering from the cells within the tissue samples.

### Cell Culture and Lysate Preparation

The epithelial ovarian cancer cell lines CP20, OV90, and OVCAR4 were routinely cultured in RPMI 1640 media containing 10% FBS and 1% penicillin‐streptomycin at 37 °C with 5% CO_2_. Cell lysates were prepared by using RIPA buffer (#BP‐115, Boston Bioproducts, Ashland, MA, USA) containing proteinase inhibitor (Pierce, Appleton, WI, USA) (1:100, v/v) according to the manufacturer's protocol.

### Protein Quantification

The protein quantification in cell lysate was performed using the BCA protein assay according to the manufacturer's protocol (Pierce BCA protein assay kit, cat. 23 250, Thermo scientific, Grand Island, NY 14 072 USA). In brief, each standard (BSA) at known concentrations ranging from 0.125–2 mg mL^−1^, and every sample were loaded into a separate well of a clear, flat‐bottomed 96‐well microplate. 10 µL volumes of all standards and samples were tested in triplicate. 90 µL of BCA working reagent (A + B) was added to each well and the plate was incubated for at least 30 min at 37 °C. After cooling to room temperature, the absorbance of all samples and standards was measured at 562 nm on a CLARIOstar plate reader (BMG Labtech, Ortenberg, Germany) to determine protein concentration.

### Determination of Saturating Protein Amount for Protein‐NP Complexation

NP‐protein complexes were made by mixing various amounts (5, 10, 25, 50, 100, and 200 µg) of OV90 protein lysates for 24 h with end‐to‐end mixing. UV−vis and DLS measurements were then conducted on the complexes. Following end‐to‐end mixing, an aggregation test was performed by adding 10% NaCl solution to the nanoconjugate solution and allowed to mix for 15 min. UV−vis spectra measurements were again conducted on the same NP‐protein complexes. Changes in absorbance, and shift in *λ*_max_ were then calculated.

### Detection of IGFBP2 Protein

IGFBP2 was detected in ovarian cancer patient plasma using a human IGFBP2 quantitative ELISA kit following the manufacturer's protocol (#DGB200, R&D Systems, Minneapolis, MN). In addition, IGFBP2 in the protein coronas formed by mixing each ovarian cancer patient's plasma, cell lysate, or conditioned media (200 µg of each) with 50 µg of GNPs for 18 h with end‐to‐end rotation at 4 °C, as previously described,^[^ ^11^ ^]^ were also determined by western blotting.

### Suppression of IGFBP2 by Gold Nanoparticles and Determination of PTEN‐Mediated Signaling Pathway

OV90 cells were grown in 100 mm culture dishes overnight and were then incubated with GNPs (25 µg ml^−1^) in non‐FBS‐containing media for 48 h. After washing, cell lysates were prepared as previously reported^[^ ^3^ ^]^ and then, the expression of mTOR, PTEN, AKT, and IGFBP2 in lysates was assessed by western blotting.

### Determination of Tumor‐Associated Signaling Pathway by a Combined Treatment of Gold Nanoparticles and Dual Kinase Inhibitor

Ovarian cancer cells (OV90 and OVCAR4) were treated with 0.75% DMSO, GNPs (25 µg ml^−1^), or PI‐103 at doses of 0.5, and 1.0 µM with or without GNPs. After 48 h, the expression of mTOR and S6K in lysates was determined by western blotting.

### Immunoblotting

For immunoblotting, cell lysates or ovarian cancer patient plasmas were incubated at 100 °C for 10 min in Laemmli buffer containing *β*‐mercaptoethanol, and the denatured cell lysates were separated on 10% tris‐glycine SDS–polyacrylamide gel electrophoresis gels before transfer to polyvinylidene difluoride membranes. Membranes were blocked using 5% BSA for 30 min at room temperature before incubation with primary antibody in 5% BSA overnight at 4 °C. Primary antibodies were rabbit anti‐IGFBP2 (1:1000), rabbit anti‐mTOR (1:1000), rabbit anti‐PTEN (1:1000), rabbit anti pS6K (1:1000), rabbit anti AKT S473(1:1000), and rabbit anti‐GAPDH (1:10 000). Following three washes with TBST (Tris Buffered Saline with 0.1% Tween20), membranes were incubated with secondary antibody at a concentration of 1:10000 for 2 h at room temperature before development with appropriate reagents.

### Cell Viability/Growth Assays

Ovarian cancer cells (CP20, OV90, and OVCAR4) were grown in 96‐well‐plates at a density of 5000 cells per well for 24 h, and then were incubated with specified doses of PI‐103, GNPs, GNPs plus PI‐103, 0.75% DMSO, or left untreated. After 48 h, cell viability/growth was assessed by counting the cells using a hemocytometer. For dose optimization of PI‐103, ovarian cancer cells (OV90 and OVCAR4) were treated with PI‐103 at various doses (0.5, 1.0, 5.0, 50, 100, and 200 µM), with 0.75% DMSO, or were not treated (NT). For cell growth inhibition assay, Ovarian cancer cells (OVCAR4 and CP20) were either treated with PI‐103 at various doses (0.5, 1.0, 5.0 µM) or pretreated with GNPs and then treated with the same doses of PI‐103. DMSO (0.75%) and GNPs (25 µg ml^−1^) served as their corresponding controls.

### Animals

Animal procedures involving NOD/SCID mice were performed by the Patient‐Derived Xenograft and Preclinical Therapeutics (PDX‐PCT) Core facility at the Oklahoma Medical Research Foundation (OMRF) and approved by the OMRF's IACUC. NOD/SCID (stock no. 0 01303) mice were purchased from the Jackson Laboratory (Bar Harbor, ME). PDXs used for this study were developed by the PDX‐PCT core facility. PDXs were generated from high‐grade serous ovarian tumors from patients of Stephenson Cancer Center at OUHSC; patients gave informed consent under a protocol approved by the OUHSC Institutional Review Board. Animals were subcutaneously implanted into the left flank with viable PDX fragments using routine procedures.^[^ ^3^ ^]^ Briefly, the mouse was anesthetized with isoflurane. The surgical site on the left flank was cleared from hairs using an electric shaver. A povidone‐iodine swab‐stick was used to sterilize the surgical area, povidone‐iodine was then removed with 70% ethanol. A 5 mm incision was made with scissors. One tumor fragment was inserted into the ovary, and the incision was closed by suturing and a wound clip; the wound clip was removed 10 days after surgery. After the tumor volume reached ≈100 mm^3^ (35 to 49 days), mice were randomized. Mice were monitored weekly for the development and progression of tumor and symptoms of physical distress or illness; body weights were also recorded weekly. Mice with established tumors of ≈100 mm^3^ were randomized and treated with GNPs as specified. Tumor dimensions were measured with a vernier caliper, and tumor volumes were calculated. Mice were treated for 5 weeks. Mice reaching SIACUC‐defined end points were euthanized by CO_2_ inhalation and necropsied. Upon completion of the experiment, tumors from all mice were collected and snap‐frozen in liquid nitrogen or fixed for downstream analyses.

Female athymic nude mice (NCr‐nu/nu; 5 to 6 weeks old) were purchased from Charles River (Delaware, Newark, USA). All mice were kept under specific pathogen‐free conditions in facilities that were approved by the American Association for Accreditation of Laboratory Animal Care and in accordance with all current regulations and standards of the U.S. Department of Agriculture, U.S. Department of Health and Human Services, and National Institutes of Health. The protocol was approved by the University of Oklahoma Health Sciences Center (OUHSC) Institutional Animal Care and Use Committee (IACUC). One set of mice received GNPs at a dose of 300 µg either intravenously or intraperitoneally. After 24 h, mice were sacrificed and liver, spleen, kidneys, heart, lungs, brain, ovary, and pancreas were collected. The GNP content of these tissues was analyzed by INAA.^[^ ^3^, ^7^ ^]^ The second set of mice received 100, 200, or 300 µg of GNPs every 2 days for 14 days, and were then sacrificed and tissues were collected and analyzed as above. The plasma from all mice was also collected and stored at −80 °C. Body weights of all mice were recorded every 2 days.

### Angiogenic Arrays

For the angiogenic assay (ARY022B, R&D Systems, Minneapolis, MN), 30 µg of protein derived from each ovarian cancer patient plasma was used and analyses were performed according to the manufacturer's protocol as described previously.^[^ ^10^ ^]^

### AST and ALT Assays

Plasma samples from GNP‐treated or control mice were thawed and analyzed. Alanine Transaminase (ALT) (#,) and Aspartate Aminotransferase (AST) (#ab105135, Abcam) were determined by colorimetric assays according to the manufacturer's instructions.

### Immunohistochemistry

Mouse tissues were fixed in 10% formalin, embedded in paraffin, and sectioned (4 µm) using a Leica multistainer (ST5020) following standard protocols. Briefly, tissue sections were deparaffinized in sequential treatments in 100% xylene, 100% ethanol, and 100% water and were then stained with H&E, Sirius Red, *α*‐SMA, CD31, NG2, or TUNEL. For staining with Ki67, *α*‐SMA, CD31, and NG2, antigen retrieval was obtained by heating the deparaffinized tissue sections in citrate buffer (pH 6) for 10 min at 95 °C. Sections were then blocked with protein block and stained with the appropriate antibodies at the specified titers overnight at 4 °C: Ki67 (1:50), *α*‐SMA (1:50), CD31 (1:50), pHHB3 (1:300), IGFBP2 (1:100), and NG2 (1:100). The ABC system (Vector) was used to detect the protein according to the manufacturer's protocol. For the detection of CD31 and NG2 positive cells, fluorescence‐labeled antibodies were used at a dilution of 1:5000. For TUNEL staining, the deparaffinized tissue sections were incubated with the in situ Cell Death Detection Kit, AP (Roche Diagnostics GmbH, Manheim, Germany) according to the manufacturer's protocol. For Sirius red (cat. ab150681, Abcam) staining, the deparaffinized tissue sections were stained according to the manufacturer's protocol. Images were captured by using a Nikon Eclipse Ni microscope

Tumor tissues were fixed in 10% formalin for 24 h, embedded with paraffin, sectioned at 4 µm thickness, and mounted on positively charged slides. Slides were deparaffinized and rehydrated in an automated Multistainer (ST5020, Leica, Wetzlar, Germany). The slides were then transferred to the BOND‐III IHC Stainer (Leica) for stepwise incubation with Epitope Retrieval Solution (pH 6, AR9961, Leica) for 20 min at 100 °C, 5% goat serum (01‐6201, ThermoFisher, MA) for 30 min at 25 °C, Peroxidase Block (RE7101, Leica) for 10 min at 25 °C, and primary antibody rabbit anti‐IGFBP2 (1:100, #3922, Cell Signaling), rabbit anti‐pHH3 (1:300, 369A‐14, Cell Marque, CA) or rabbit anti‐CD31 (1:50, #77 699, Cell Signaling, MA) overnight at 4 °C. The Bond Polymer Detection System (DS 9800, Leica) was then applied. The slides were dehydrated and mounted with MM 24 (3 801 120, Leica). To quantify IGFBP2 staining, H‐score was estimated with the formula H = 0 × (% cells on the whole section at intensity 0) + 1 × (% cells at intensity 1) + 2 × (% cells at intensity 2) + 3 × (% cells at intensity 3), where the staining intensity of 0 = negative, 1 = weak, 2 = moderate and 3 = strong. To quantify pHH3 staining, the number of positively stained cells in 200x microscopic field were counted with ImageJ (NIH, MD). Three fields from each section were counted. To quantify micro‐vessel density (MVD) as illustrated by CD31 staining, three microscopic images of 20x fields in each section that have the greatest micro‐vessel density (hotspots) were taken. Any red staining of cell or cell cluster that was separate from adjacent micro‐vessels was considered a single, countable vessel. All tumors were counted.

### Statistics

All results are derived from at least three individual experiments, unless stated otherwise, and are reported as means ± SD. The statistical significance of differences between groups was determined using one‐way analysis of variance (ANOVA) followed by multiple comparisons test via Prism Pad software or student *t*‐test. A *P* value of <0.05 was considered to be significant. In addition, we used the linear mixed model to analyze the tumor volume change in 10 days by 5 groups for Figure 5 using a using multiple testing corrected significant threshold of 0.005 (0.05/10). We used the Kruskal‐Wallis test to analyze the tumor weight at end point among 5 groups using multiple testing corrected significant threshold of 0.005 (0.05/10).

## Conflict of Interest

The authors declare no conflict of interest.

## Author contributions

P.M. supervised the project. P.M. and M.N.H. conceived the project, designed the study, and wrote the manuscript. M.N.H. performed the experiments and data analysis. M.N.H. and L.W. prepared orthotropic PDX model mice and performed the therapeutic study on this model under the guidance of P.M. and M.B. S.K.D.D, G.R. C.E. S.A. S.D, C.X, V.S, A.D., and S.W. helped in reproducing the data for cellular mechanisms. J.D.R. measured the gold content in various tissues. R.B. helped in designing and conducting experiments (microscopy) and was involved in all discussions. All authors reviewed the manuscript.

## Supporting information

Supporting Information

## Data Availability

The data that support the findings of this study are available from the corresponding author upon reasonable request.
