# Supplementary material for: Gold Nanoparticles Disrupt the IGFBP2/mTOR/PTEN Axis to Inhibit Ovarian Cancer Growth
Source: Adv Sci (Weinh). 2022 Sep 14;9(31):2200491. doi: 10.1002/advs.202200491 (PMC9631030; doi:10.1002/advs.202200491)
Supplement: Supplementary file 1 — Supporting Information [file ADVS-9-2200491-s001.pdf]

Supplementary Information

Gold Nanoparticles Disrupt the IGFBP2/mTOR/PTEN Axis to Inhibit Ovarian Cancer Growth

Contents

Supplementary Figure 1-5 and Figure Legends

Supplementary Figure 1 | Characterization of gold nanoparticles (GNPs).

Supplementary Figure 2 | Immunohistopathological analysis of patient-derived ovarian cancer tissue.

Supplementary Figure 3 | Immunofluorescence analysis of patient-derived ovarian cancer tissue.

Supplementary Figure 4 | 20 nm GNPs accumulation in GNPs treated tumor model mice.

Supplementary Figure 5 | Protein corona characterization around 20 nm GNPs.

Supplementary Table 1: Pairwise comparisons of group and time interaction between groups.

Supplementary Table 2: Pairwise comparisons of end point tumor weight between groups.

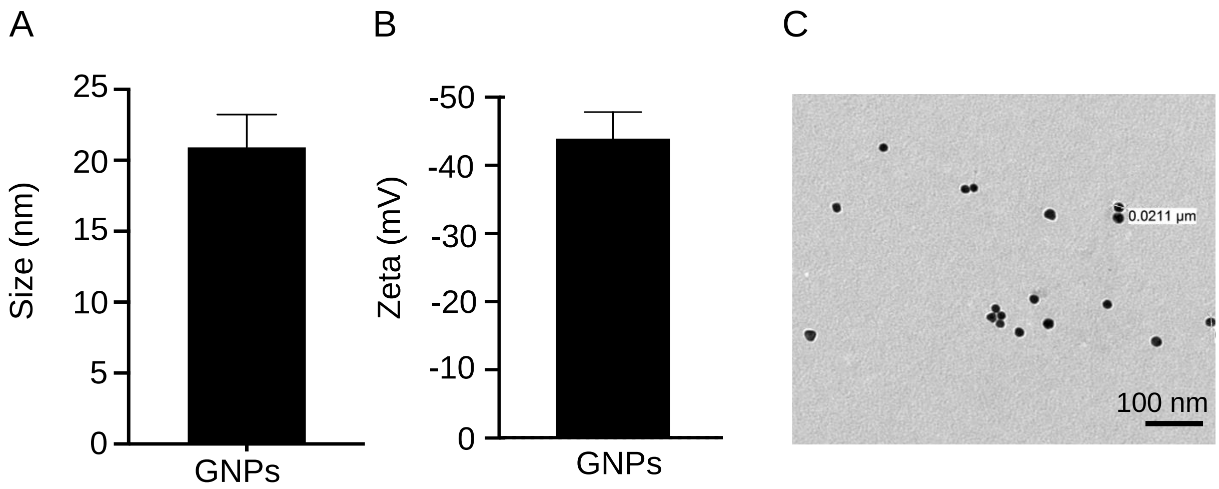

Supplementary Figure 1. GNPs characterizations. (A-B) The size and surface charge of GNPs were determined by DLS and zeta potential measurements (n=4). (C) TEM micrographs of as-synthesized GNPs exhibiting spherical particles of ~ 20 nm diameter. Scale bar represents 100 nm.

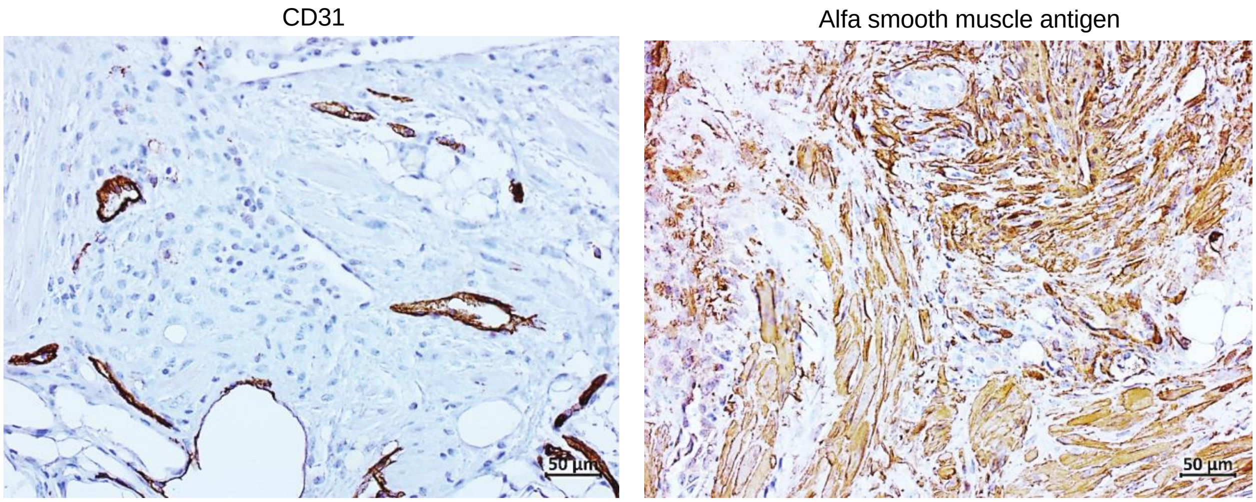

Supplementary Figure 2. Immunohistopathological analysis of patient-derived ovarian cancer tissue. The tumorigenic properties of patient-derived ovarian cancer tissue (PDX-098) were evaluated in terms of determining expression levels of alpha-smooth muscle actin (α-SMA) and endothelial cells marker (CD31) via immunostaining. Scale bar represents 50 µm.

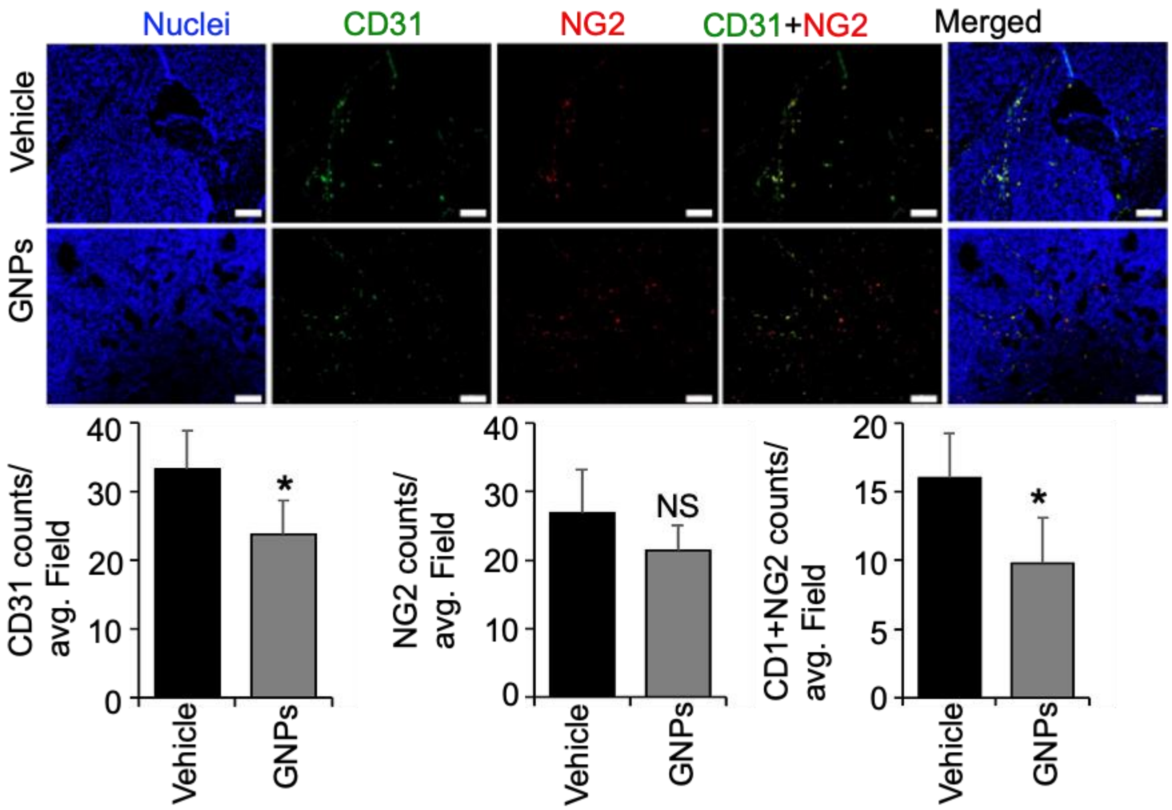

Supplementary Figure 3. Immunofluorescence analysis of patient-derived ovarian cancer tissue. Representative tumor tissues from GNP and vehicle treated mice were immunostained by using CD31 and NG2 antibodies and fluorescence labeled secondary antibodies were used for visualization; nuclei were stained with DAPI (blue). The CD31 and NG2 positive cells are green and red respectively; colocalization appears as yellow. The intensities of CD31 and NG2 positive cells and their colocalization were quantified using ImageJ and analyses were performed using student *t*-test. *$P \leq 0.05$, n=15.

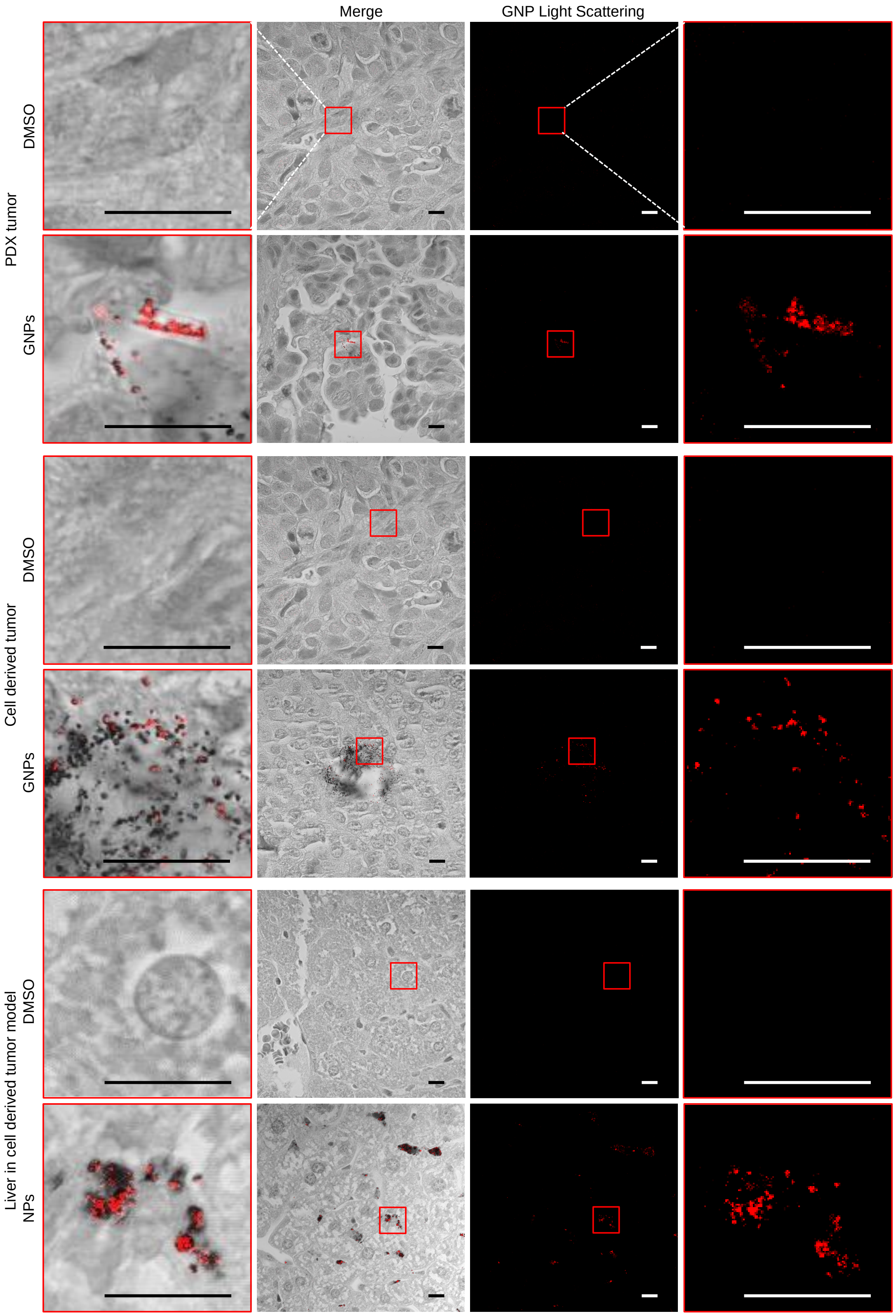
Merge
GNP Light Scattering
PDX tumor
DMSO
GNPs
Cell derived tumor
DMSO
GNPs
Liver in cell derived tumor model
DMSO
NPs

Supplementary Figure 4: GNPs accumulation in GNP treated tumor model mice. (A) Tumor tissues from GNP-treated and PBS-treated PDX model mice were collected, sliced and were observed under a Zeiss LSM 880 CLSM. Scale bar factors are 20X - 2.4089 pixels/µm and 63X - 7.5879 pixels/µm. (B) Tumor and liver tissues from GNP-treated and DMSO-treated ovarian cancer cell derived model mice were collected, sliced and images were taken using this same microscope and conditions.

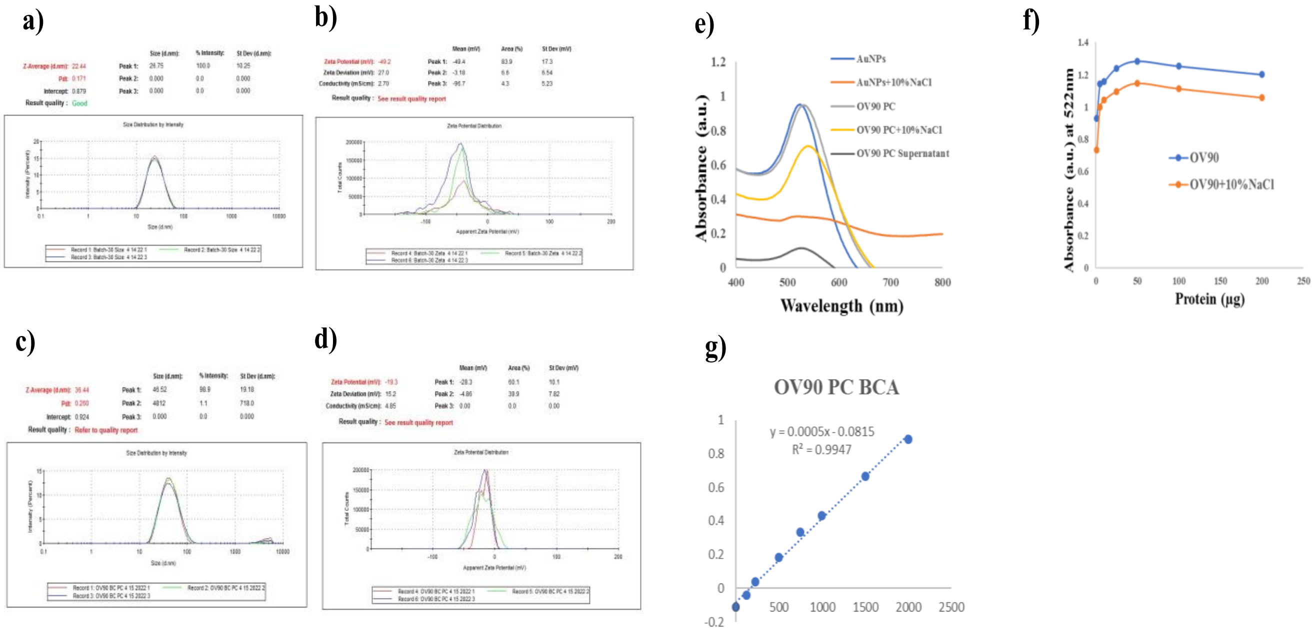

Supplementary Figure 5: Protein corona characterization around 20 nm GNPs. (a-d) Size and zeta potential of 20 nm GNPs before and after incubation with OV90 lysate proteins. (e, f) UV-Visible spectra of bare GNPs and GNP-protein corona before and after incubation with 10 % NaCl solution. (g) Estimation of protein content in the corona by BCA method.

Supplementary Table 1.

	PI-103 (Twice a week)	PI-103 (Daily)	GNP	PI-103 + GNP
DMSO	50.14 (6.72e-10)	90.56 (<2e-16)	107 (<2e-16)	119.62 (<2e-16)
PI-103 (Twice a week)		40.42 (4.37e-8)	56.86 (1.89e-14)	69.48 (<2e-16)
PI-103 (Daily)			16.44 (0.021)	29.06 (9.98e-5)
GNP				12.62 (0.076)

*Each cell presented the estimated difference (group in row – group in column) and its p-value of testing the difference was 0.

Supplementary Table 2:

	PI-103 (Twice a week)	PI-103 (Daily)	GNP	PI-103 + GNP
DMSO	0.442	0.021	0.0006	0.0003
PI-103 (Twice a week)		0.066	0.002	0.0003
PI-103 (Daily)			0.161	0.029
GNP				0.867

*Each cell presented the Wilcoxon rank sum exact test p-value.
